# TERT Promoter Mutations Lead to High Transcriptional Activity under Hypoxia and Temozolomide Treatment and Predict Poor Prognosis in Gliomas

**DOI:** 10.1371/journal.pone.0100297

**Published:** 2014-06-17

**Authors:** Chen Chen, Sheng Han, Lingxuan Meng, Zhonghua Li, Xue Zhang, Anhua Wu

**Affiliations:** 1 Research Center for Medical Genomics, Key Laboratory of Medical Cell Biology, Ministry of Education, College of Basic Medical Science, China Medical University, Shenyang, Liaoning, China; 2 Department of Neurosurgery, the First Affiliated Hospital of China Medical University, Shenyang, Liaoning, China; 3 Department of Medical Genetics, Peking Union Medical University, Peking, China; University Hospital of Navarra, Spain

## Abstract

**Objective:**

This study explored the effects of *telomerase reverse transcriptase* (*TERT*) promoter mutations on transcriptional activity of the *TERT* gene under hypoxic and temozolomide (TMZ) treatment conditions, and investigated the status and prognostic value of these mutations in gliomas.

**Methods:**

The effect of *TERT* promoter mutations on the transcriptional activity of the *TERT* gene under hypoxic and TMZ treatment conditions was investigated in glioma cells using the luciferase assay. *TERT* promoter mutations were detected in 101 glioma samples (grades I–IV) and 49 other brain tumors by sequencing. *TERT* mRNA expression in gliomas was examined by real-time PCR. Hazard ratios from survival analysis of glioma patients were determined relative to the presence of *TERT* promoter mutations.

**Results:**

Mutations in the *TERT* promoter enhanced gene transcription even under hypoxic and TMZ treatment conditions, inducing upregulation of *TERT* mRNA expression. Mutations were detected in gliomas, but not in meningiomas, pituitary adenomas, cavernomas, intracranial metastases, normal brain tissues, or peripheral blood of glioma patients. Patients with *TERT* promoter mutations had lower survival rates, even after adjusting for other known or potential risk factors, and the incidence of mutation was correlated with patient age.

**Conclusion:**

*TERT* promoter mutations were specific to gliomas. *TERT* promoter mutations maintained its ability of inducing high transcriptional activity even under hypoxic and TMZ treatment conditions, and the presence of mutations was associated with poor prognosis in glioma patients. These findings demonstrate that *TERT* promoter mutations are novel prognostic markers for gliomas that can inform prospective therapeutic strategies.

## Introduction

The *telomerase reverse transcriptase* (*TERT*) gene encodes the catalytic subunit of telomerase, which is normally not expressed by postmitotic somatic cells. Telomere deficiency has been linked to cellular aging and cancer, while upregulation of human *TERT* gene expression has been associated with the acquisition of stem cell-like properties, including immortalization [1]. Mutations in the *TERT* promoter that create a novel binding site for T-cell factor (TCF) transcription factors, thereby increasing *TERT* gene transcription, have been identified in cutaneous melanoma. Individuals with these mutations have increased cancer susceptibility, as demonstrated in studies of familial melanoma [2], [3]. TCF transcription factors have important roles in developing and adult brains [4], [5], [6], [7] as the main effectors of the canonical Wnt/β-catenin signaling pathway, which is dysregulated in various types of cancers [8].

Glioblastoma is the most frequently occurring brain tumor among adults. The standard treatments for glioblastoma are surgery, radiation therapy, and temozolomide (TMZ) chemotherapy; however, the high incidences of tumor recurrence and mortality make it imperative to devise more effective strategies to manage glioblastoma through improved diagnostic and treatment measures [9], [10], [11]. For grade II astrocytoma, the prognosis of certain patients is markedly poor. We hypothesized that some pathological molecular changes leading to constant abnormal expression of mitosis related genes may contribute to the poor prognosis. In this study, the effect of *TERT* promoter mutations was examined in glioma cells, and the mutation status of the promoter was analyzed in glioma patients with respect to glioma progression and patient survival.

## Materials and Methods

### Patients and Tissue Samples

The 101 glioma and corresponding peripheral blood samples analyzed in this study were obtained from the Chinese Glioma Genome Atlas (CGGA) specimen bank at the First Hospital of China Medical University. The samples consisted of 28 primary glioblastomas (P; grade IV), 34 anaplastic astrocytomas (AA; grade III), 34 astrocytomas (A; grade II), and five pilocytic astrocytomas (PA; grade I). In addition, 22 meningiomas, 17 pituitary adenomas, six intracranial metastases, four cavernomas, and two normal brain tissue samples were included, the latter obtained from patients that had undergone surgery for primary epilepsy. All patients underwent surgical resection from January 2006 to December 2007. The histological diagnoses were established and verified by two neuropathologists according to the 2007 World Health Organization classification guidelines. All high-grade glioma (grades III and IV) patients received post-operative, standard radiation therapy and chemotherapy according to the Stupp protocol [10]. All astrocytoma (grade II) patients received post-operative TMZ chemotherapy. This study was approved by the institutional review boards of the First Hospital of China Medical University. All tissue samples were immediately flash frozen in liquid nitrogen after resection and written informed consent for future research usage of the sample was obtained from every patient. Before the experiment, the percentage of tumor cells in each sample was assessed by staining a frozen section with hematoxylin and eosin. Only samples with more than 80% tumor cells were selected for analysis.

### Genomic DNA Extraction

Frozen tissue samples were thawed on ice and lysed with 490 µl lysis buffer containing 20 mM Tris-Cl (pH 8.0), 5 mM EDTA (pH 8.0), 400 mM NaCl, and 1% (w/v) SDS, to which 10 µl proteinase K (10 mg/ml) was added for digestion at 37°C for 12 h. Genomic DNA was purified from the lysate by phenol/chloroform extraction, and resuspended in 50 µl TE buffer (pH 8.0). Genomic DNA was extracted from peripheral blood samples using a universal genomic DNA extraction kit (TaKaRa Bio Inc., Shiga, Japan).

### Mutational Screening of the *TERT* Promoter

The *TERT* core promoter was amplified by PCR using previously published primers [2], [3]. The PCR was carried out in a 30 µl reaction volume containing approximately 60 ng genomic DNA, 0.3 µl TaKaRa LA Taq (TaKaRa Bio Inc.), 15 µl 2× GC Buffer I, 4.8 µl dNTP mixture (2.5 mM each), and 1.5 µl each primer (10 µM). The PCR conditions were as follows: initial denaturation at 95°C for 5 min, followed by 35 cycles of 94°C for 30 s, 62°C for 30 s, 72°C for 30 s, and final elongation at 72°C for 7 min. The PCR products were gel purified and sequenced on an ABI PRISM 3730XL Genetic Analyzer (Applied Biosystems, Foster City, USA).

### Analysis of *TERT* mRNA Expression

*TERT* expression levels were determined by quantitative reverse transcriptase PCR (qRT-PCR). Total RNA was extracted from glioma tissue samples using Trizol reagent (Invitrogen, Carlsbad, USA) according to the manufacturer’s instructions. Reverse transcription of 1 µg total RNA was performed to generate cDNA using the PrimeScript RT reagent Kit with gDNA Eraser (TaKaRa Bio Inc.) in a 20 µl reaction volume. The qRT-PCR reaction volume of 20 µl contained 0.5 µl each of primers hTERT-qRT-F (5′-ACT GGC TGA TGA GTG TGT ACG TCG T-3′) and hTERT-qRT-R (5′-ACC CTC TTC AAG TGC TGT CTG ATT CC-3′) (10µM), 10 µl SYBR Premix Ex Taq (TaKaRa Bio, Inc.), and 4 µl cDNA, with four replicates per sample. Reactions were performed on a Rotor-Gene 6000 real-time rotary analyzer (Corbett Life Science, Sydney, Australia) at 95°C for 10 min, followed by 40 cycles of 95°C for 10 s, 60°C for 15 s, and 72°C for 20 s. *TERT* mRNA levels were normalized to a glyceraldehyde-3-phosphate dehydrogenase fragment. Relative expression level was determined using the comparative ΔΔCT method with a calibration curve generated from cDNA from normal brain tissue. Experiments were repeated at least three times.

### Plasmid Construction

The 474 bp human TERT promoter (−391 to +83) was amplified by PCR from normal germline DNA using primers TERT-F (5′-GGG GTA CCC TGG CGT CCC TGC ACC CTG G-3′) and TERT-R (5′-CCC AAG CTT ACG AAC GTG GCC AGC GGC AG-3′. The amplified fragment was inserted into the KpnI/HindIII sites of the pGL3-Basic Vector (Promega, Madison, USA) to generate the pTERT-WT construct. Using the same strategy, the two mutation constructs, including −124 C>T and −146 C>T mutaion, were generated by amplifying genomic DNA fragments from TERT mutated tumor tissues. All constructs were verified by Sanger sequencing.

### Cell Culture, Transfection, and Drug Treatment

U87 glioma cells were cultured in high glucose DMEM, supplemented with 10% fetal bovine serum and 1% penicillin/streptomycin, at 37°C in an atmosphere of 5% CO_2_. Constructs (1 µg) were transfected, along with the pRL-TK Renilla luciferase vector (10 ng) to normalize the transfection efficiency, into U87 glioma cells growing in a 24-well plate at 80–90% confluence, using 2.0 µl Lipofectamine 2000 reagent (Invitrogen). Cells were subjected to one of the following treatments 24 h after transfection. *TMZ treatment*. TMZ was dissolved in dimethyl sulfoxide (DMSO; Sigma-Aldrich, Saint Louis, USA); cells were treated with 50 µM TMZ for 24 h, at a final DMSO concentration of less than 0.1% (v/v). Cells in the control group were treated with the equivalent volume of DMSO. *CoCl_2_ treatment*. CoCl_2_·6H_2_O (Sigma-Aldrich) solution (100 mM) was made using ddH_2_O, and used to mimic hypoxic conditions. Cells were treated with 150 µM CoCl_2_ for 24 h, with cells exposed to ddH2O serving as a control. *Hypoxia treatment*. Cells were placed overnight in an incubator in an atmosphere of 1% O_2_, 5% CO_2_, and 94% N_2_ at 37°C. Control cells grown in normoxic conditions were maintained in an incubator with 5% CO_2_, 20% O_2_, and 75% N_2_ at 37°C.

### Luciferase Reporter Assay

Following the above-described treatments, cells were washed with 1× PBS and assayed for luciferase activity according to the Dual Luciferase Reporter assay protocol (Promega). Each treatment, along with the corresponding control, was administered in parallel to cells in four wells. Relative luciferase activity was calculated as the ratio of firefly to Renilla luciferase.

### Statistical Analysis

Cox proportional hazards models were used to calculate hazard ratios (HRs) of patient survival according to *TERT* promoter mutation status, unadjusted or adjusted for age, sex, tumor extension, tumor size, preoperative Karnofsky performance status (KPS), resection, and tumor grade. To adjust for potential confounds, age, tumor size, and preoperative KPS were used as continuous variables, and all other covariates were used as categorical variables. Four variables were dichotomized: tumor extension (one lobe vs. more than one lobe), resection (gross total vs. subtotal), tumor grade (III vs. IV), and *TERT* promoter status (mutated vs. wild-type). There were three age categories (<45, 45–60, and ≥60 years) and four categories for tumor location (frontal, temporal, fronto-temporal, and parietal lobes). A Kaplan-Meier analysis was performed to determine the distribution of overall survival (OS) times, and a log-rank test was used to compare the distributions. The χ^2^ test was used to examine associations between categorical variables. The *t* test was performed to compare the means for age, tumor size, and preoperative KPS. All analyses were performed using SPSS 13.0 (SPSS Inc., Chicago, USA), and a two-tailed *P* value <0.05 was considered significant.

## Results

### *TERT* Promoter Mutations are Associated with Gliomas

**Figure 1:**
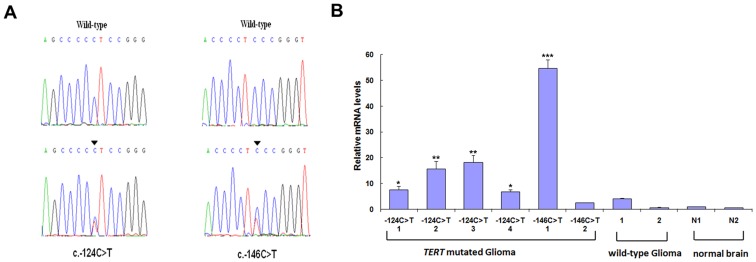
TERT promoter mutation and mRNA expression. A: DNA sequence chromatograms of glioma tissue samples with mutations in the *TERT* promoter. Single nucleotide transitions (C>T) were observed at −124 bp or −146 bp from the ATG translation start site of the *TERT* gene. B: Detection of *TERT* mRNA levels in glioma tissue samples with and without *TERT* promoter mutations, and in two normal brain tissue samples. Gliomas with mutations include four samples with the −124 C>T mutation, and two with the −146 C >T mutation. Wild-type gliomas lacked mutations in *TERT* promoter region. N1 and N2 are normal brain tissues. *TERT* gene expression was significantly higher in mutated *TERT* promoter glioma samples compared to normal tissue samples, while this was not observed in wild-type *TERT* promoter glioma samples. The reference, N1, was considered as having a value of one. *P<0.05; **P<0.01; ***P<0.001 (compared with N1).

Among 152 tumor samples, *TERT* promoter mutations were found only in gliomas, and not in meningiomas, pituitary adenomas, cavernomas, intracranial metastases, normal brain tissues, or in peripheral blood samples (Fig. 1A). Of 101 glioma samples, mutations were detected in 45 (44.6%), including 33 (73.3%) that were –124 C>T and 12 (26.7%) that were –146 C>T. Clinical, pathological, and molecular features of the glioma cases were examined according to mutation status (Table 1). *TERT* promoter mutations were significantly associated with patient age (*P* = 0.004). Moreover, compared to tumors with wild-type *TERT* promoters, tumors with *TERT* promoter mutations were more likely to be of a high pathological grade (*P* = 0.039).

**Table 1:** Clinical and Molecular Characteristics According to TERT Promoter Mutational Status in 101 Glioma Cases.

Clinical or MolecularFeature	All Cases			TERT Promoter						*P*
				Wild-type			Mutated			
	No. (%)			No. (%)			No. (%)			
Total No. of patients	101			56 (55.4%)			45 (44.6%)			
Sex										0.841
Male	54 (53.47%)			29 (53.70%)			25 (46.30%)			
Female	47 (46.53%)			27 (57.45%)			20 (42.55%)			
Age, years										0.000
Mean±SD	46.99±15.47			42.02±16.29			53.07±11.83			
<45	48 (47.52%)			33 (68.75%)			15 (31.25%)			*0.004*
45–60	41 (40.59%)			21 (51.22%)			20 (48.78%)			
≥60	12 (11.88%)			2 (16.67%)			10 (83.33%)			
Tumor location										0.649
Frontal lobe	40 (39.60%)			24 (60.00%)			16 (40.00%)			
Temporal lobe	27 (26.73%)			12 (44.44%)			15 (55.56%)			
Fronto-temporal lobe	8 (7.92%)			4 (50.00%)			4 (50.00%)			
Parietal lobe	7 (6.93%)			4 (57.14%)			3 (42.86%)			
Tumor extension										0.486
One lobe	77 (76.24%)			41 (53.25%)			36 (46.75%)			
More than one lobe	24 (23.76%)			15 (62.50%)			9 (37.50%)			
Tumor size, cm^3^										0.267
Mean±SD	185.76±148.37			198.78±181.18			169.18±88.10			
Preoperative KPS										0.480
Mean±SD	76.73±10.37			76.07±10.47			77.56±10.20			
Resection										1.000
Gross total	69 (68.32%)			38 (55.07%)			31 (44.93%)			
Subtotal	32 (31.68%)			18 (56.25%)			14 (43.75%)			
Tumor grade										0.041
I	5 (4.90%)			5 (100.00%)			0 (0.00%)			
II	34 (33.70%)			22 (64.70%)			12 (35.30%)			
III	34 (33.70%)			18 (52.94%)			16 (47.06%)			
IV	28 (27.70%)			11 (39.30%)			17(60.70%)			
Low-grade (I–II)	39 (38.60%)			27 (69.20%)			12 (30.80%)			*0.039*
High-grade (III–IV)	62 (61.40%)			29 (46.80%)			33 (53.20%)			

### Mutations in the *TERT* Promoter Increase mRNA Expression in Gliomas

*TERT* promoter mutations create new binding motifs for Ets/TCF transcription factors close to the transcription start site, and in reporter assays, have been shown to cause increases in transcriptional activity of up to two-fold [3]. Thus, the effect of *TERT* promoter mutations on *TERT* mRNA expression in gliomas was examined by qRT-PCR. The presence of mutations −124 C>T and −146 C>T was associated with increased expression of *TERT* mRNA in gliomas (Fig. 1B).

### Mutations in the *TERT* Promoter Predict Patient Survival in High-grade Gliomas

**Figure 2:**
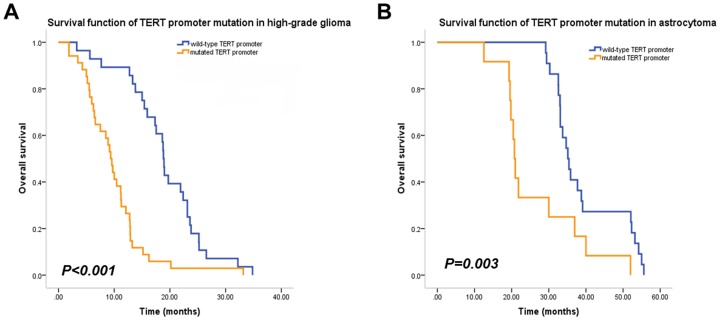
Survival function of TERT promoter mutation in glioma patients. A: Kaplan-Meier analysis showing the OS of high-grade glioma patients with wild-type (blue line) and mutated (orange line) *TERT* promoters. The difference in OS was significant between the two groups. B: Kaplan-Meier analysis showing the overall survival (OS) of low-grade glioma (*i.e.*, astrocytoma) patients with wild-type (blue line) and mutated (orange line) *TERT* promoters. The difference in OS was significant between the two groups.

The impact of *TERT* promoter mutations on patient survival in high-grade gliomas was assessed. Compared to patients with wild-type *TERT* promoters, those with *TERT* promoter mutations had higher overall mortality (univariate HR 2.735; 95% CI: 1.611 to 4.641; *P*<0.001) (Table 2). In the multivariate Cox model adjusted for potential predictors of patient outcome, TERT promoter mutations were associated with a significant increase in overall mortality (HR 4.148; 95% CI: 1.973 to 8.721; *P*<0.001) (Fig. 2A).

**Table 2:** Univariate and Multivariate Analysis of Different Prognostic Parameters for Overall Survival of High-grade Glioma Patients.

Variable	Univariate analysis			Multivariate analysis		
	*P*	HR	95% CI	*P*	HR	95% CI
Sex	0.944	0.982	0.586 to 1.644	0.837	0.942	0.532 to 1.666
Age	0.063	1.017	0.999 to 1.035	0.760	1.003	0.981 to 1.026
Extension	0.093	0.593	0.322 to 1.090	0.532	0.792	0.382 to 1.644
Size	0.595	1.000	0.998 to 1.001	0.898	1.000	0.998 to 1.002
Preoperative KPS	0.662	1.006	0.980 to 1.032	0.621	1.007	0.980 to 1.035
Resection	0.699	1.113	0.648 to 1.911	0.271	1.453	0.748 to 2.823
Grade (III *vs* IV)	0.017	1.415	0.845 to 2.368	<0.001	3.652	1.785 to 7.473
TERT promoter mutation	<0.001	2.735	1.611 to 4.641	<0.001	4.148	1.973 to 8.721

The two-year survival was also examined with respect to *TERT* promoter mutations. Survival at 2 years was 31.03% (9/29) for patients with wild-type *TERT* promoters, compared to 3.03% (1/33) for those with mutations (*P* = 0.001). The −124 C>T and −146 C>T mutations were also analyzed separately; however, there was no significant difference between these mutations in terms of mortality rate (data not shown). *TERT* promoter mutations were examined across the strata of age and tumor grade, and were found to be associated with poor OS in all subgroups.

### Mutations in the *TERT* Promoter Predict Patient Survival in Astrocytomas

The effect of *TERT* promoter mutations on survival was examined in astrocytoma (grade II) patients. Compared to patients with wild-type *TERT* promoters, those with mutated promoters had higher overall mortality (univariate HR 3.208; 95% CI: 2.114 to 4.868; *P*<0.001) (Fig. 2B and Table 3). In the multivariate Cox model adjusted for potential risk factors, *TERT* promoter mutations were associated with a significant increase in overall mortality for grade II gliomas (HR 3.058; 95% CI: 1.886 to 4.958; *P*<0.001).

**Table 3:** Univariate and Multivariate Analysis of Different Prognostic Parameters for Overall Survival of Astrocytoma Patients.

Variable	Univariate analysis			Multivariate analysis		
	*P*	HR	95% CI	*P*	HR	95% CI
Sex	0.700	0.923	0.615 to 1.385	0.813	0.948	0.612 to 1.470
Age	0.005	1.019	1.005 to 1.032	0.822	1.002	0.985 to 1.019
Extension	0.410	0.823	0.517 to 1.309	0.553	0.847	0.490 to 1.464
Size	0.802	1.000	0.999 to 1.001	0.766	1.000	0.998 to 1.001
Preoperative KPS	0.899	1.001	0.983 to 1.020	0.832	1.002	0.982 to 1.023
Resection	0.023	2.108	1.363 to 3.260	0.009	1.846	1.168 to 2.917
TERT promoter mutation	<0.001	3.208	2.114 to 4.868	<0.001	3.058	1.886 to 4.958

### Increased Transcriptional Activity Under Hypoxia and TMZ Treatment are Associated with Mutations in the *TERT* Promoter

**Figure 3:**
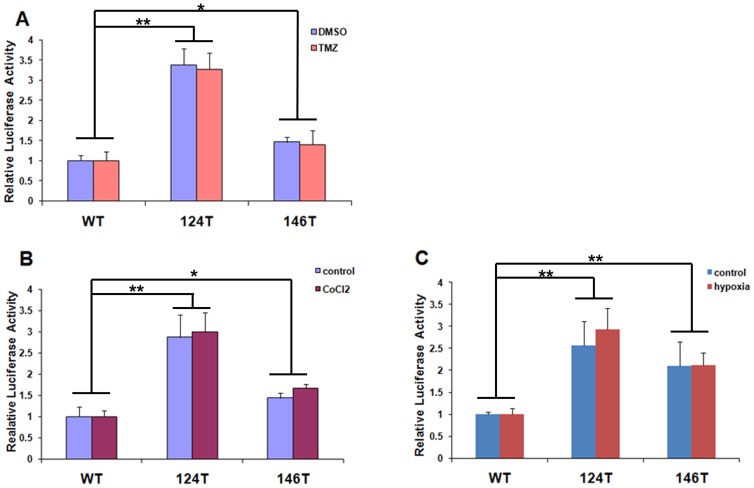
Luciferase reporter assays for transcriptional activity from the *TERT* core promoter with −124 C>T or −146 C>T mutations compared to wild-type promoter in U87 cell lines. Cells were treated with TMZ, or cultured under hypoxic conditions through CoCl_2_ treatment or exposure to 1% O_2_. WT, wild-type; 124T, −124 C>T mutation; 146T, −146 C>T mutation. (A) Compared to wild-type, 124T and 146T displayed significantly higher *TERT* promoter activity in the presence of TMZ. No significant difference was observed between the DMSO (control) and TMZ (50 µM) treatment groups. (B) Compared to wild-type, 124T and 146T displayed significantly higher *TERT* promoter activity under hypoxic conditions. No significant difference was observed between control and CoCl_2_ treatment groups (B); similar results were observed when hypoxia was induced by exposure to 1% O_2_ (C). The means of four measurements per experimental group are shown; error bars indicate standard deviation. *P<0.05; **P<0.01.

A luciferase reporter assay was used to test whether transcriptional activity from the mutated *TERT* promoter changed as a result of hypoxia and TMZ treatment in the U87 glioma cell line. Compared to the wild-type *TERT* promoter, −124 C>T and −146 C>T mutations induced increases of approximately 3- and 1.5-fold, respectively, in transcriptional activity (Fig. 3). Cells treated with 50 µM TMZ or the equivalent volume of DMSO had nearly identical luciferase activity, suggesting that *TERT* transcription in glioma cells was not blocked by TMZ (Fig. 3A). Cells treated with CoCl_2_ had luciferase activity levels comparable to control cells, indicating that chemically induced hypoxia had no effect on *TERT* transcription (Fig. 3B). Similarly, no difference in luciferase activity was observed between cells exposed to normoxic and hypoxic conditions (Fig. 3C). Taken together, these results indicate that mutations in the *TERT* promoter lead to persistently high transcriptional activity even under hypoxic or TMZ treatment conditions.

## Discussion

Glioblastoma is the most common type of primary brain tumor, yet the prognosis for glioma patients remains poor. Since 2005, a standard protocol of radiotherapy combined with TMZ chemotherapy has been used to treat high-grade gliomas, with a 2-year survival rate of about 26.5% [10]. Predicting tumor recurrence is a significant challenge for glioma treatment. Although it has been reported that methylation of the O6-methylguanine-DNA methyltransferase (MGMT) gene promoter predicts patient response to radio- and chemotherapy as well as prognosis [11], [12], [13], [14], [15], it did not apply to all patients, and other studies were unable to find a correlation between MGMT methylation and prognosis [16]. This is complicated by the fact that MGMT methylation status can change between the first surgery for newly diagnosed glioblastomas, and the second surgery for a recurring tumor [17]. Therefore, the identification of new prognostic biomarkers for glioblastoma is critically important. Such markers could also be used to screen for individuals who are at a higher risk for tumor recurrence, so that treatment approaches can be tailored to each patient, thereby potentially sparing patients from some of the inevitable complications that accompany the current forms of therapy.

The results of the present study demonstrate that mutations in the *TERT* gene promoter are associated with decreased survival in glioma patients (Fig. 2 and Table 2). The role of telomerase in tumorigenesis is well-established in many types of cancer, and high telomerase activity is linked to high tumor malignancy [18] and chemo-resistence [19], [20], [21]. The activation of telomerase is tightly regulated at the transcriptional level, with several studies indicating that *TERT* transcription is the rate-limiting step in telomerase expression [22]. As such, *TERT* mRNA expression level has been suggested as a biomarker for gliomas [23]. However, this may be unsuitable for a few reasons. First, the expression of *TERT* is influenced by many environmental factors [24], [25], and individual differences may also undermine the prognostic value. Second, tumor samples contain a mixture of tumor and other cell types, such as immune and vascular endothelial cells among others, making it difficult to measure *TERT* expression levels in tumor cells exclusively. Moreover, due to the heterogeneity of gliomas themselves, there is no baseline for making comparisons of *TERT* expression. Genomic mutations represent more reliable markers than gene expression. In melanomas with *TERT* promoter mutations, *TERT* is stably expressed at a high level [3]. Similarly, in gliomas with mutations in the *TERT* promoter, high levels of *TERT* mRNA were detected (Fig. 1B), possibly resulting from the specific mutations. The sustained upregulation of *TERT* expression implies a continuous activation of telomerase, which could contribute to the immortalization of glioma cells and their resistance to therapeutic measures. We proposed that the TERT promoter mutation may maintain its ability of inducing high transcription activity even the microenvironment is changed, this was supported by the finding that mutated *TERT* promoter constructs induced high levels of transcriptional activity under conditions of hypoxia and TMZ treatment (Fig. 3).

*TERT* promoter mutations were detected only in gliomas and not in other types of brain tumor (Fig. 1A), indicating that the identified mutations could serve as glioma-specific biomarkers to predicting tumor occurrence or recurrence. Given that the mutations were significantly correlated with high pathological grade (Table 1), they may actually contribute to glioma malignancy. It is well-established that hypoxia usually occurred with glioma progression [26], [27], and the present study found that high levels of *TERT* gene transcription were maintained from the mutated promoter during hypoxia [28].

The traditional methods for assessing the pathology of aggressive gliomas have limitations. For instance, due to the heterogeneity of gliomas, there may be biases in sample collection that could interfere with diagnosis and the selection of a treatment strategy. Screening for *TERT* promoter mutations could circumvent this bias to offer individualized, more effective treatments for glioblastoma patients. Additionally, the poor prognosis of grade II astrocytomas with *TERT* promoter mutations suggests that these tumors can be classified into an aggressive subtype. There is an ongoing debate about whether grade II astrocytoma patients should receive chemotherapy alone or in combination with radiotherapy. Distinguishing different astrocytoma subtypes based on *TERT* promoter mutation status could provide a molecular basis for pursuing more intensive treatments, although the present results indicated that high-grade glioma patients harboring the mutations did not respond well to chemo- and radiotherapy.

It was also found that *TERT* promoter mutation frequency was significantly correlated with patient age (Table 1), consistent with previous reports that age predicts poor prognosis in glioblastoma [29]. The median OS in elderly patients after diagnosis is typically less than 1 year [30], which was confirmed by this study. Taken together, the findings suggest that *TERT* promoter mutations could be a causal factor in the mortality of elderly patients. In consistence with our study, Vinagre et al and Killele et al also reported that *TERT* promoter mutation was associated with age and prognosis in large glioma patient population [31], [32].

The results of this study demonstrate that *TERT* promoter mutations–specifically, −124 C>T and −146 C>T–can be used alone or possible in conjunction with other mutations, such as the isocitrate dehydrogenase 1 mutation and MGMT methylation, as a prognostic biomarker and indicator of pathological grade of gliomas. This can provide a means for making more accurate predictions of treatment in patients. Future studies will focus on elucidating the mechanisms by which *TERT* promoter mutations confer resistance to TMZ treatment, and on examining the association between these mutations and others that promote glioma progression.
